# Combinations of Energy‐based Devices plus isotretinoin for management of acne and acne scars: A systematic review

**DOI:** 10.1111/jocd.16407

**Published:** 2024-06-06

**Authors:** Yidan Xu, Hao Wang, Linghong Guo, Michael R. Hamblin, Xiang Wen

**Affiliations:** ^1^ Department of Dermatology, West China Hospital Sichuan University Chengdu China; ^2^ Laboratory of Dermatology, Clinical Institute of Inflammation and Immunology, Frontiers Science Center for Disease‐related Molecular Network, West China Hospital Sichuan University Chengdu China; ^3^ Laser Research Centre, Faculty of Health Science University of Johannesburg Doornfontein South Africa

**Keywords:** acne vulgaris, isotretinoin, laser treatment, phototherapy, systematic review

## Abstract

**Background and Objective:**

A 6‐month interval between systemic isotretinoin (ISO) and the initiation of energy‐based interventions has been recommended, due to concerns about keloid formation and delayed wound healing. While this postponement goes against the current trend of early intervention for acne scarring. This systematic review evaluates the efficacy, safety, and patient satisfaction of combinations of ISO with energy‐based devices (EBD).

**Study Design/Methods and Materials:**

PubMed, Embase, Web of Science, Cochrane Library, and Cochrane Central Register of Controlled Trials were comprehensively searched up to April 2023 according to PRISMA guidelines. Two independent reviewers screened the titles and abstracts to select articles. The quality of the literature was assessed for each study design.

**Results:**

A total of 16 studies addressing the efficacy and safety of energy‐based modalities combined with ISO were identified, including six randomized controlled trials (RCTs), two case series, seven cohort studies, and one case report. ISO combinations with intense pulsed light (IPL), fractional ablative CO_2_ laser, pulsed dye laser (PDL), non‐ablative fractional laser (NAFL) and fractional microneedle radiofrequency (FMRF) have been tested for improving acne severity, acne scarring and erythema.

**Conclusion:**

The current evidence does not justify delaying the use of EBDs for patients who have recently undergone or are currently receiving ISO treatment. Evidence‐based treatments such as PDL, NAFL, and FMRF etc. are suggested relatively safe and effective in treating acne and acne scarring.

## 1 INTRODUCTION

Acne vulgaris is a prevalent inflammatory skin disorder affecting nearly 10% of the global population, and ranks as the eighth most common disease worldwide.^1^, ^2^ This condition is almost universal among young people, with a peak incidence in postpubescent teens.^3^ Besides its primary lesions, there are other secondary complication, such as erythema, hyperpigmentation, and post‐acne scars.^4^, ^5^ The inflammation of active acne can lead to scarring as a result of an imbalance between the breakdown of the matrix and the production of collagen.^6^ Acne scar is not only a significant cosmetic issue, but is also associated with an increased risk of psychological problems, including depression and poor overall quality of life.^7^ Thus, prompt and adequate treatment is essential for managing acne scarring.^4^

The pathogenesis of acne has been attributed to four interrelated factors: inflammation, excessive sebum production, follicular hyper‐keratinization, and colonization with *Cutibacterium acnes* bacteria.^8^ Oral isotretinoin (ISO), which targets the tetrad of acne pathophysiological features is the most effective treatment currently available for severe and recalcitrant acne. However, there is concern that systemic ISO can lead to some adverse effects, including dryness of skin, dry eye syndrome, cheilitis, and also has a risk of teratogenicity.^9^, ^10^ Despite multiple treatment regimens being available, the inconvenience in use, prolonged course of therapy, and worries about antibiotic resistance or other side effects often lead to poor compliance, thus impeding successful treatment. Therefore, combination therapy with an accelerated treatment course and more pronounced clinical benefits is urgently needed.

Energy‐based devices (EBDs) have been increasingly investigated as an alternative in the management of acne vulgaris. Studies have indicated that various forms of EBDs, such as intense pulsed light (IPL), carbon dioxide (CO_2_) laser, non‐ablative fractional laser (NAFL), and pulsed dye laser (PDL) can potentially manage moderate‐to‐severe acne.^11^ Multiple studies have demonstrated the prompt interventions play a vital role in managing acne vulgaris,^2^, ^12^ and individuals who suffer from severe acne and are currently using or have recently finished taking ISO are most likely to benefit from EBDs treatments to reduce the risk of scarring.^13^ However, it has been traditionally recommended that any cosmetic procedure, including lasers, radiofrequency, chemical peels, etc., should be postponed until at least 6–12 months after the cessation of systemic ISO treatment. Notably, it was based upon a few early case reports of delayed wound healing and keloid formation,^14^, ^15^, ^16^ and this conservative strategy has recently been challenged by several expert consensus opinions.^17^, ^18^, ^19^ According to the consensus recommendation, there was insufficient evidence in favor of a 6‐month procedural delay between finishing ISO treatment and receiving non‐ablative laser or similar treatment.^19^, ^20^ Considering the increasing trend of combining EBDs and systemic ISO, we performed a systematic review to evaluate the efficacy and safety of concomitant systemic ISO with EBDs in acne patients.

## 2 METHODS

The study was carried out following the preferred reporting items for Systematic Reviews and Meta‐Analyses (PRISMA) guidelines (Figure 1).

**FIGURE 1:**
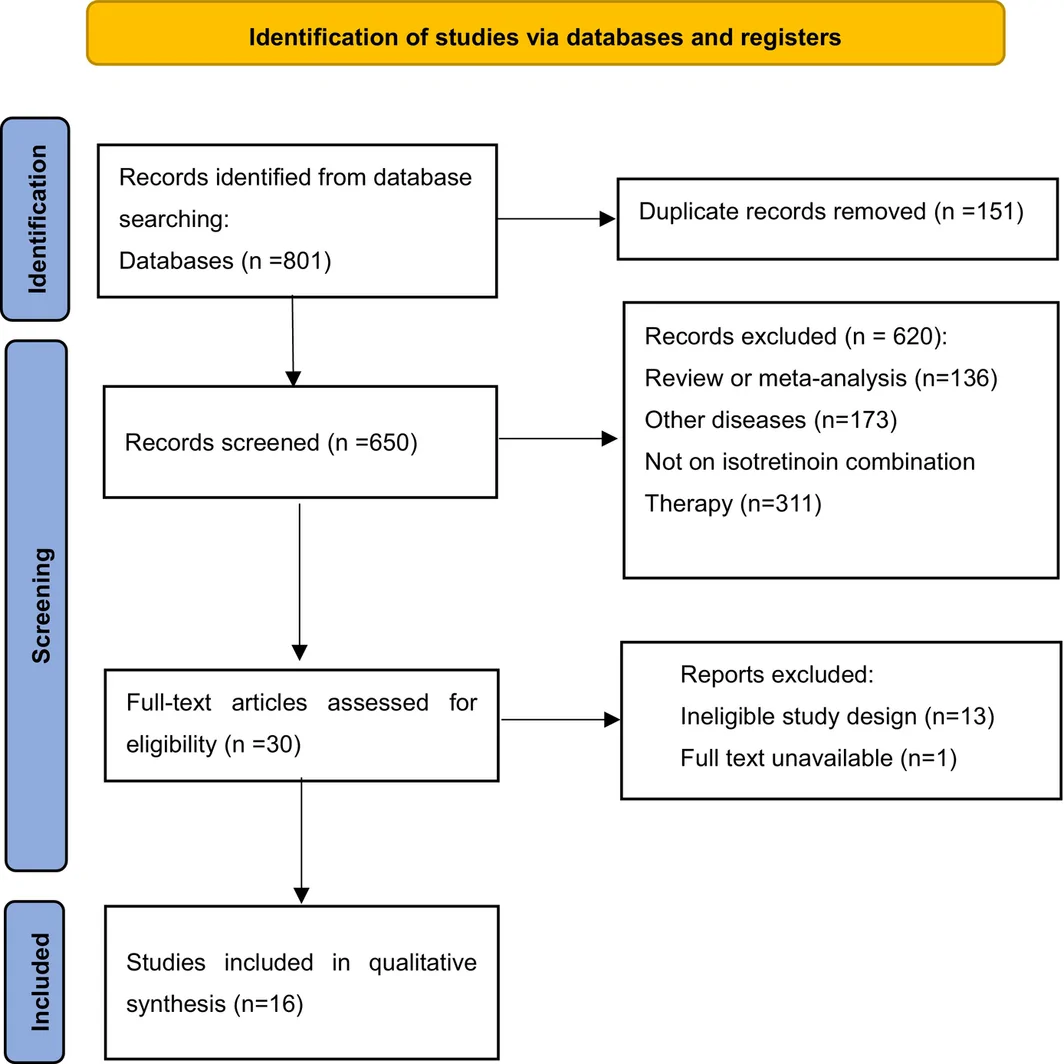
Flow diagram of literature search and study selection.

### 2.1 Search strategy and study selection

We searched for published articles concerning the concomitant use of ISO with energy‐based modalities for treating acne, in PubMed, Embase, Web of Science, Cochrane Library databases, and Cochrane Central Register of Controlled Trials up to April 2023. We also manually searched the reference lists of the original articles and relevant reviews. The search strategies are shown in Table 1.

**TABLE 1:** Summary of search terms.

Search strategy component and Number	Query
Patients	
1	Acne vulgaris
Interventions	
2	MeSH descriptor isotretinoin explode all trees
3	MeSH descriptor phototherapy explode all trees
4	MeSH descriptor photochemotherapy explode all trees
5	MeSH descriptor photosensitizing agents explode all trees
6	MeSH descriptor photolysis explode all trees
7	MeSH descriptor laser explode all trees
Merge	
8	3 or 4 or 5 or 6 or 7
9	2 and 8
10	1 and 9

### 2.2 Inclusion and exclusion criteria

The inclusion criteria were:

1. Study type: randomized or non‐randomized controlled trials (RCTs), case cohorts, case reports, and case series;
2. Participants: patients with a diagnosis of acne vulgaris of any severity defined by any classification system;
3. Intervention: light, laser or radiofrequency therapy combined with oral ISO;
4. outcomes: change in acne severity from the baseline based on the Investigator Global Assessment (IGA) scale; participant self‐reporting global improvement in acne, change or percentage change in mean total lesion count from the baseline assessed by the investigator; adverse events assessed by the investigator; changes in quality of life and participant satisfaction with treatment.

Exclusion criteria: any study not focusing on the efficacy or side effects of combination therapy; studies not in English.

### 2.3 Data extraction

Data including study design, subject characteristics, intervention types, and outcomes were extracted from the original articles. The following intervention parameters were gathered: dosage of ISO, modality devices, parameters of energy‐based modalities, and interval and sessions employed for separate studies. In terms of outcome, we specifically recorded changes in acne severity, acne scar improvement, erythema recovery, adverse events, and patient satisfaction (Tables 2 and 3).

**TABLE 2:** Summary of studies.

Author, year	Study design	Level of evidence	Patient characteristics	Device	Treatment regimen	Control regimen	Assessed parameters
Gold et al. (2020)^30^	Prospective cohort study	Level 3	46 subjects18 males, 28 femalesAge: 18–30 yearsFitzpatrick type: I–III	Nd: YAG lasers	Parameters: 1064 nm, 21 J/cm^2^, 650 ms pulse width, 6 mm spot sizeInterval: 2 weeksIsotretinoin dosage: 0.2–0.3 mg/kg/dDuration: 6 months	Not available	• Acne severity• Patients' satisfaction• Adverse events
Gao et al. (2020)^25^	Retrospective cohort study	Level 4	60 subjects 20 males, 40 females Age: 20–40 years Fitzpatrick type: III–IV	NAFL	Parameters: 1560 nm, 60 mJ, 150 spots/cm^2^ for lesions; 40–45 mJ, 200 spots/cm^2^ for whole face Interval & Sessions: 6 weeks, 3 sessions Isotretinoin dosage: 1 mg/kg/d (first 2‐4 W); 0.5 mg/kg/d (next 12–14 W) Duration: 16 weeks	Not available	• Lesion counts reduction • Scars improvement • Patients' satisfaction
Saluja et al. (2018)^28^	Single blind, randomized split‐face controlled trial	Level 2	10 females Age: 18–36 years Fitzpatrick type: I–III	Erbium‐doped NAFL	Parameters: 1550 nm, 35–40 mJ/MTZ, eight passes per treatment Interval & Sessions: 4 weeks, 3 sessions Isotretinoin cumulative dosage: 125.0–325.0 mg/kg Interval between ISO and laser: 1–4 weeks	Untreated side	• Scarring improvement • Patients' satisfaction • Adverse events
Hann et al. (2010)^31^	Prospective cohort study	Level 3	35 subjects Age: 16–48 years Fitzpatrick type: II–V	Infrared fractional laser	Parameters: 1550 nm, 50%–75% of conventional densities Isotretinoin dosage: 10 mg/d Interval between ISO and laser: 1 month	Not available	• Scarring improvement • Adverse events
Xia et al. (2018)^27^	Single blind, randomized split‐face controlled trial	Level 2	18 females Age: 18–38 years	Er:glass fractional laser	Parameters: 1550 nm, 20 mJ/cm^2^, 100–169 spots per area Interval & Sessions: 1 month, 3 sessions Isotretinoin dosage: 0.14–0.23 mg/kg/d Duration: 9 months	Not available	• Acne severity • Scars improvement • Patients' satisfaction • Pain evaluation

Author, year	Study design	Level of Evidence	Patient characteristics	Device	Treatment regimen	Control regimen	Assessed parameters
Leal el al. (2011)^32^	Retrospective cohort study	Level 4	30 subjects 11 males, 19 females	Fractionated Erbium laser	Parameters: 50 to 70 mJ, 4–5 level, 3–7 sessions Isotretinoin dosage: 20 mg/d Duration: On ISO for 11.4 ± 4.6 months before laser treatment	Patients with acne not on ISO were treated with fractionated erbium laser	• Adverse events
Moradi et al. (2021)^30^	Prospective Open‐label controlled trial	Level 3	148 subjects 27 males, 121 females Age: 17–44 years	NLite and Er: YAG laser	NLite parameters: 3 J/cm^2^, 7 mm Spot size, three sessions Er: YAG parameters: 0.6–0.7 J/cm^2^, 3.5 to 5 mm spot size, 5 weekly sessions Isotretinoin dosage: 1 mg/kg Duration: 6 months	Aged match patients with the same Lesions Isotretinoin dosage: not available Duration: not available	• Patients satisfaction • Post‐inflammatory hyperpigmentation • Adverse events
Kim et al. (2014)^33^	Retrospective cohort study	Level 4	20 subjects 9 males, 11 females Age: 15–38 years Fitzpatrick skin type III‐V	Ablative fractional CO_2_ laser	Parameters: 30–50 mJ, 0.96–1.67 ms, 30 W, 100–200 MTZ/cm^2^, 6.4% 12.7% of coverage Interval: more than 1 month Isotretinoin dosage: 10–40 mg/d Interval between ISO and laser: Concomitant or 4 weeks	Not available	• Scarring improvement • Adverse events
Kim et al. (2020)^33^	Retrospective cohort study	Level 4	71 subjects 33 males, 38 females Age: 19–38 years Fitzpatrick types: III‐V	FMRF + ablative fractional CO_2_	Parameters: RF: 8 level, 25 MTZ/cm^2^, 2.0–2.8 mm depth; CO2: 100 mJ, 100 spots/cm^2^, 15.6% coverage, 1168 mm depth Interval: average 3.31 sessions Isotretinoin dosage: 20 mg/d, 120–150 mg/kg Duration: 4–6 weeks	Not available	• Acne severity • Scarring improvement • Skin texture improvement
Kalashnikova et al. (2020)^34^	Case report	Level 4	1 male Age: 16 years	ND: YAP/KTP+ Er: YAG laser	Parameters: first stage: 1079/540 nm ND: YAP/KTP; second stage: 2940 nm Er: YAG laser Interval & Sessions: ND: YAP/KTP: 2–3 weeks interval, 4 sessions; Er: YAG laser: 4 weeks interval, 9 sessions Isotretinoin dosage: 16 mg/d		• Quality of life • Adverse events

**TABLE 3:** Summary of results and side effects.

Author, year	Key findings	Efficacy evaluation	Complications
Russo et al. (2021)^26^	Acne severity: 1–3 grade improvement at week 12 Patients' satisfaction: 75% were very satisfied and 25% were satisfied	IGA scale 5‐point Likert scale	Transient erythema or hyperpigmentation Dryness No severe adverse effects
Youbao et al. (2022)^25^	GAGS improvement: 70.88% (ISO + DPL) vs. 62.12% (ISO) (*p* = 0.0009) Papule counts improvement: 61.58% (ISO + DPL) vs. 43.33% (ISO) (*p* < 0.0001) Comedone counts improvement: 63.15% (ISO + DPL) vs. 43.30% (*p* = 0.0008) DPL co‐treatment showed better improvement is TEWL, EI and MI Patients' satisfaction: higher satisfaction score with DPL treated side than the control side (*p* < 0.0001)	GAGS Lesion counts Transepidermal Water Loss (TEWL) Erythema index (EI) Melanin index (MI)	Mild to moderate dry cheilitis, 2 cases of dry eyes Tolerable stinging sensation DPL treatment 2 transient erythema, 1 edema No pigmentation or scarring
Li et al. (2020)^35^	GEA improvement: 2.8 ± 0.7 to 1.8 ± 0.8 (ISO + IPL) vs. 2.7 ± 0.7 to 2.3 ± 0.4 (ISO) (*p* < 0.05) Total lesion improvement: 79.2% (ISO + IPL) vs. 65.2% (ISO) (*p* < 0.01) DLQI: 4.7 ± 2.2 (ISO + IPL) vs. 6.3 ± 1.9 (ISO) (*p* < 0.05) Patients' satisfaction: higher satisfaction score with IPL combined group than the control group (*p* < 0.01)	Global Evaluation Acne (GEA) grade Lesion counts Dermatology life quality index (DLQI) and visual analog scale satisfaction	Mild erythema and tolerable pain in IPL group Dry lips observed in both groups 1 case of blisters in IPL group No severe adverse event
Ibrahim et al. (2021)^24^	GAGS improvement: 76.1% (ISO + PDL) vs. 60.8% (ISO) (*p* = 0.04) Acne erythema improvement: 1.91 ± 0.85 to 0.57 ± 0.66 (ISO + PDL) vs. 1.74 ± 0.81 to 1 ± 0.67 (*p* = 0.031) CADI: 8.96 ± 2.36 to 1.39 ± 1.16 (ISO + PDL) vs. 8.52 ± 2.35 to 2.43 ± 1.56 (ISO) (*p* = 0.016)	Global acne grading system (GAGS) 4‐point grading of acne erythema Cardiff Acne Disability Index (CADI)	6 subjects suffered from flare in ISO group, none in the ISO + PDL group 20 subjects suffered from dryness in ISO group, 5 in the ISO + PDL group No pigmentation or scarring
Gold et al. (2020)^36^	Acne severity: 1.8 ± 0.2 to 0.5 ± 0.4, 72.2% reduction (*p* < 0.01) DLQI: 10.1 ± 1.3 to 2.8 ± 1.2, 72.3% (*p* < 0.01)	Investigators Global Assessment (IGA) scale DLQI	None
Xia et al. (2018)^27^	ECCA improvement: 10.4 to 3.5 (ISO + Er: glass fractional laser) vs. 10.6 to 5.8 (ISO) (*p* < 0.05) Scars improvement: Significant recovery of superficial (*p* < 0.05) and deep boxcar atrophic (*p* < 0.01) Patients' satisfaction: 11.1% very satisfied, 66.7% satisfied, and 22.2% neutral	Leeds acne‐grading scores Echelle d'evaluation clinique des cicatrices d'acne (ECCA) grading scale Visual analogue scale	
Gao et al. (2020)^25^	Improvement on inflammatory papules: 86.7% (ISO + 1565 nm NAFL) vs. 73.3% (ISO) Boxcar scars improvement: 60.0% vs. 80.0% Patient satisfaction: 86.7% vs. 66.7%	Improvement rates Satisfaction rate VAS scoring system	Transient edema, erythema, scab and hyperpigmentation No scar or keloid formation
Huan et al. (2023)^29^	ECCA improvement: 32.21% (ISO + FxPico) vs. 9.22% (ISO) GAGS improvement: 12.6 ± 3.4 to 7.8 ± 2.8 (ISO + FxPico) (*p* < 0.0001) vs. 12.6 ± 3.3 to 10.1 ± 3.0 (ISO) (*p* = 0.00766), Lesion counts: better effect observed in ISO + FxPico group Both monotherapy and combination with FxPico significantly decreased TEWL. No significant change in both EI and MI DLQI: Highest satisfaction rate in ISO + FxPico	ECCA GAGS Lesion count TEWL, MI, EI DLQI	Dryness Tolerable pain Transient erythema and edema No severe adverse effects
Saluja et al. (2018)^28^	Acne scarring improvement: 40% showed acne scar improvement of 1–25%, 50% had an improvement of 26–50%, and 10% showed 51–75% improvement Patient satisfaction: all satisfied	2‐point scale for wound healing 4‐point grading scale for improvement in acne scarring and satisfaction	Mild erythema and edema No hypertrophic scars nor keloids
Hann et al. (2010)^31^	Acne scarring improvement: 80% of moderate to excellent improvement; 20% of some improvement or no effect	5‐point grading scale	1 case of mild acneiform eruption 3 cases of scaly itchy foci No hypertrophic scars or keloid formation
Moradi et al. (2021)^30^	Patient satisfaction: all satisfied	Visual analog scale	Moderate to severe cheilitis Post inflammatory Hyperpigmentation: 2.80% vs. 2.67%
Kim et al. (2014)^37^	Acne scarring improvement: moderate to much improved SGA compared to baseline at 6 months in most patients	Scar Global Assessment score (SGA) Patients' self‐assessment	None
Kim et al. (2020)^33^	Acne scarring improvement: 3.19 ± 0.70 to 1.53 ± 0.53 (ISO + FMRF + CO_2_) vs. 2.89 ± 0.74 to 1.46 ± 0.51 (FMRF + CO_2_) (*p* < 0.05)	Grade of Acne Severity Scar Global Assessment score (SGA)	3 cases of acral dermatitis related to ISO Transient erythema and edema No delays in wound healing
Durso et al. (2019)^38^	Significant erythema improvement in all patients	Not available	None
Leal el al. (2011)^32^	No difference in erythema, edema, and scabbing during the recovery process	Not available	None
Kalashnikova et al. (2020)^34^	Quality of life: DLQI decreased from 24 to 6 Stable remission of inflammation, aesthetic improvement of scars	Dermatology life qusality index (DLQI)	None

### 2.4 Assessment of study quality

The methodological quality of the included studies was assessed by two independent investigators using appropriate tools for different study designs. RCTs that compared ISO alone with a combination of ISO with energy‐based modalities were evaluated by Cochrane risk‐of‐bias tool. The JBI critical appraisal checklist was used for cohort studies, case series and report.^21^, ^22^, ^23^ Any discrepancy was discussed and resolved by a senior reviewer (Table 4).

**TABLE 4:** Evidence quality evaluation of the included RCTs.

Author, year	Random sequence generation	Allocation concealment		Performance bias	Completeness of results	Selective reporting
		Operator & subjects	Outcome evaluator			
Ibrahim et al. (2021)^24^	Sufficient	Single‐blinded	Blinded	None	Sufficient	None
Youbao et al. (2022)^25^	Sufficient	Non‐blinded (Split‐face)	Blinded	None	Sufficient	None
Li et al. (2020)^26^	Sufficient	Non‐blinded	Blinded	Unclear	Sufficient	None
Xia et al. (2018)^27^	Unclear	Non‐blinded (Split‐face)	Blinded	Unclear	Six dropouts	Unclear
Saluja et al. (2018)^28^	Sufficient	Single‐blinded (Split‐face)	Blinded	None	Sufficient	None
Huan et al. (2023)^29^	Sufficient	Non‐blinded (Split‐face)	Blinded	None	Sufficient	None

## 3 RESULTS

We identified 650 non‐duplicated search results, of which 30 studies were considered potentially relevant according to their titles and abstracts. Eventually, a total of 16 studies were identified.

## 4 EARLY INTERVENTION OF ACNE VULGARIS

Several seminal consensuses suggested there isn't enough evidence to postpone laser or other EBD with those received ISO. This delay goes against the current trend of early intervention for scar formation.^12^, ^17^, ^18^

### 4.1 ISO combined with blue light

Russo et al. studied the efficacy of combining blue light (150 mW/cm^2^, peak wavelength of 440–460 nm) with low‐dose oral ISO. All enrolled patients experienced a substantial improvement in acne symptoms, especially inflammatory lesions. When evaluated at week 52, the IGA for acne severity reached and was maintained at 0 (3–4 score at baseline) in patients receiving fluorescent light energy and oral ISO therapy.^26^

### 4.2 ISO combined with pulsed light

In a RCT including 47 patients currently on ISO, a biweekly treatment with 420 nm IPL (0–15 J/cm^2^, 30 ms pulse width, 10 × 20 mm spot size) was randomly assigned to individuals. After eight sessions of treatment, a significant reduction in global evaluation acne grade and total lesions count were observed in the IPL & ISO combination group. Concurrent IPL plus ISO treatment also significantly decreased the percentage of inflammatory lesions (79.2% vs. 56.5%, *p* < 0.01).^35^

Youbao et al. studied the synergistic effects of ISO combined with low‐level light therapy (LLLT) using delicate pulsed light (DPL). In their split‐face RCT, significant improvement in acne severity was observed in the DPL & ISO combination group, as suggested by lower global acne grading system (GAGS) score and reduction in lesion counts. The reduction in the number of papules, pustules, and comedones was more significant on the combination side than on the ISO alone side.^25^ The restoration of normal skin color was also observed in DPL treated side, suggesting an anti‐inflammatory effect of DPL.^25^ Noticeable improvements in inflammation were also found in Gao's study.^25^

### 4.3 ISO combined with PDL

A randomized, single‐blinded controlled trial with 46 subjects compared the clinical benefits of concurrent low‐dose ISO (0.25 mg/kg/day) plus PDL treatment, with a standard dosage of ISO (0.5 mg/kg/day). Five sessions of PDL were performed at two‐week intervals in the combination group. Parameters were: 4.5–5.5 J/cm^2^, 3 ms, and 10 mm spot size. Acne severity improved by 76.1% and 60.8% from baseline in the combination and the control group respectively, when evaluated at the six‐month follow‐up. Notably, the combination group showed significantly better GAGS scores than the standard dose group.^24^

Ibrahim et al. demonstrated that combination of PDL and systemic ISO is more effective in reducing erythema of acne.^24^ These findings were consistent with another case series which suggest 595 nm PDL treatment significantly improved generalized erythema.^38^

### 4.4 ISO combined with infrared laser

In a prospective cohort study with 46 subjects carried out by Gold et al., low‐dose ISO (0.2–0.3 mg/kg/d) combined with 1064 nm Nd: YAG facial laser treatment (21 J/cm^2^, spot diameter of 6 nm via a collimated beam) was reported to improve acne severity, (IGA score decreased from 1.8 ± 0.2 to 0.5 ± 0.4; 72.3% reduction; *p* < 0.01) after 12 sessions of Nd: YAG laser.^36^

## 5 EARLY INTERVENTION OF ACNE SCAR

### 5.1 ISO with infrared laser

A recent split‐face trial studied the efficacy of early intervention of scarring with picosecond laser. In this study, 24 patients with atrophic acne scars were treated with low‐dose oral ISO and two sessions of fractional picosecond 1064 nm Nd: YAG laser (FxPico) treatment. FxPico treatment was found to be safe and effective in improving all types of atrophic acne scars, including boxcar, rolling, and icepick scars, with significant improvements in ECCA score, skin barrier function and patients' life quality. Interestingly, boxcar scar showed the most prominent improvement after FxPico treatment.^29^

Xia J et al. designed a split‐face RCT to study the efficacy of non‐ablative factional lasers in acne patients currently on low‐dose ISO (0.14–0.23 mg/kg/d). The intervention side of the face received three sessions of low‐energy Er:glass fractional laser (1550 nm, 35–40 mJ/MTZ, eight passes per treatment), at one‐month intervals, whereas the other side of the face served as a control. After completing all sessions, the 1550 nm NAFL laser‐treated side showed a significant improvement in both superficial (*p* < 0.05) and deep boxcar atrophic scars (*p* < 0.01).^27^ Meanwhile, the 1550 nm NAFL‐treated side obtained a better improvement in comedone numbers, but the difference in the number of papules and nodules on the two sides of the face was not significant.^27^

Another similar split‐face trial with ten patients by Saluja et al. tested 1550 nm erbium‐doped NAFL (35–40 mJ/MTZ, eight passes per treatment) plus oral ISO for atrophic acne scar management. Half of the subjects displayed 26%–50% improvement in acne scars, four patients showed up to 25% improvement, and only one patient showed 51%–75% improvement.^28^

In a prospective cohort study with 35 subjects treated with a combination of ISO and fractional infrared laser, most patients reported moderate to excellent acne scar improvement. However, 20% showed no effect or only slight improvement.^31^

Gao et al. conducted a four‐arm retrospective cohort study to investigate the benefits of a combination of 1565 nm NAFL with or without pricking blood therapy (PBT) plus ISO (ISO). In this study, the NAFL + ISO arm (*n* = 15) used 1565 nm NAFL (inflammatory lesions and scars: 60 mJ, 15 spots/cm^2^; full face: 40–45 mJ, 200 spots/cm^2^) plus systemic ISO (0.5 mg/kg/d). The other three arms were treated with ISO (*n* = 15), 1565 nm NAFL (*n* = 15), or ISO + NAFL + PBT (*n* = 15). In the double‐therapy group, nine out of 15 patients showed a remarkable improvement in inflammatory papules, with an 86.7% total improvement rate, compared with 73.3% and 67.0% for the ISO alone and NAFL alone group, respectively. Meanwhile, better improvement of boxcar atrophic scars was also observed in the double‐therapy group.^25^

A patient with acne conglobatа was treated with a combination of ISO plus vascular laser 1079/540 nm ND: YAP/KTP in areas prone to hypertrophic scarring on the face and neck. Next, the 2940 nm Er: YAG laser with SMA technology was applied to the acne scars. There were four sessions with 2–3 week intervals for the vascular laser, and nine sessions of Er: YAG laser on the face, with five sessions on the back and shoulders. After 2 years of combination therapy, the patient showed a progressive improvement in inflammation and acne scars. Notably, his psychological state was positively improved.^34^

### 5.2 ISO with CO_2_ laser

A retrospective cohort study showed that ablative CO_2_ fractional laser (single or double passes with pulse energy 30–50 mJ, 0.96–1.67 ms, 30 W, density of 100–200 MTZ/cm^2^, 6.4%–12.7% of coverage) plus oral ISO showed moderate to good improvements in acne scars in the majority of patients at the 6 month evaluation.^37^ While in an earlier study of 55 patients receiving ISO alone at a dose of 0.5 mg/kg/d and 25 patients receiving fractional CO_2_ laser in combination with ISO, no statistically significant differences were observed with respect to scarring or side effects, as reported in reference.^26^

### 5.3 ISO combined with radiofrequency

A retrospective cohort study by Kim et al., applied three sessions of FMRF + CO_2_ laser in acne patients, and reported an overall significant reduction in Scar Global Assessment (SGA) scores, when comparing the initial value 3.14 ± 0.76 to the final value 1.58 ± 0.70 (*p* < 0.05). The authors also compared patients taking ISO (*n* = 43) with those not on ISO. The combination of ISO plus FMRF + CO_2_ showed significantly better improvement in the severity of acne scars (2.89 ± 0.74–1.46 ± 0.51; *p* < 0.05). Meanwhile, the number of inflammatory lesions (papules and pustules) dropped significantly from 2.63 ± 0.81 at baseline to 1.2 ± 0.41 after three sessions. There was also a better improvement in skin texture in the combination group.^33^

## 6 PATIENT SATISFACTION

Patients' satisfaction was documented in a total of six studies, and the majority reported a high level of satisfaction regardless of the grading scales used.

### 6.1 ISO combined with light therapy

Li, Y et al. demonstrated that patients were more satisfied with ISO plus 420 nm IPL treatment, compared to ISO only, as measured by DLQI (DLQI: 4.7 ± 2.2 vs. 6.3 ± 1.9, *p* < 0.05; *p* < 0.01).^35^

### 6.2 ISO combined with laser therapy

A clinical trial also measured patient satisfaction for the combination of 1064 nm laser plus ISO. They found a significant 72.3% improvement in DLQI from the initial 10.1 ± 1.3–2.8 ± 1.2 (*p* < 0.01).^36^ One study evaluated patient satisfaction using the Cardiff Acne Disability Index (CADI), which is a short questionnaire assessing four domains: patient psychological and social status, acne on the trunk, mood, as well as self‐assessment of acne severity.^39^ This study suggested that the combination of ISO plus PDL could produce better satisfaction compared to ISO alone, with a significant reduction in CADI from 8.96 ± 2.36–1.39 ± 1.16 in the combination group vs. 8.52 ± 2.35–2.43 ± 1.56 in the monotherapy group, (*p* = 0.016).^24^

There were two randomized split‐face control trials assessing patient satisfaction with a combination of NAFL plus oral ISO. In the subjects treated with 1565 nm NAFL combination therapy, 86.7% reported feeling satisfied or very satisfied, whereas only 66.7% and 60.0% reported the same satisfaction levels in the ISO or the NAFL alone groups, respectively.^25^ Likewise, in another single‐blinded RCT, almost 80% of patients felt satisfied or very satisfied with the combination therapy of 1550 nm NAFL plus low‐dose ISO.^27^

## 7 SAFETY AND TOLERABILITY

The overall evidence suggested that the combination of ISO and energy‐based procedures is safe and tolerable. The literature reported neither keloid nor hypertrophic scar formation, but only some mild and transient side effects associated with either the oral ISO or the procedures. The most common adverse effects were dryness of skin or mucosa, especially dry lips and eyes. For instance, Ibrahim et al. reported a much lower incidence of dryness symptoms in the PDL combination group, compared to patients treated with ISO alone. One patient even experienced an attack of epistaxis in the monotherapy group in this study.^24^ Moderate to severe cheilitis was also a common adverse effect observed. Additionally, three patients showed clinical signs of acral dermatitis, one subject developed mild acneiform eruption, and three suffered from scaly, itchy foci,^31^, ^33^ which were thought to be associated with systemic ISO use.

Other frequent adverse effects included mild erythema, edema, and hyperpigmentation, that all subsided within a few days. In the Xia J et al. study, more than 90% of subjects complained of transient erythema and edema after NAFL treatment.^27^ One retrospective trial suggested that both the combination therapy and oral ISO alone were equally well tolerated. There was no difference in the recovery process of erythema, edema, or post‐inflammatory hyperpigmentation.^30^, ^32^ Notably, one patient developed a 1 cm blister shortly after IPL treatment. A total of seven studies reported the duration of adverse effects, including erythema, edema, crusting, etc. The erythema and edema commonly resolved within 30 min–7 days, whereas other mild side effects subsided within 2 weeks. The pain related to the procedural modalities was also discussed. More than 60% of subjects reported mild to moderate pain when treated with IPL combined with ISO, whereas combination therapy with 1550 nm NAFL produced varying levels of pain in all patients.^24^, ^27^, ^35^ One study also reported the temporary pain score related to laser intervention, ranging from 3 to 7 points.^25^ There was no relapse reported during the follow‐up period. However, the follow‐up duration in the included articles was relatively short, varying from 1–6 months, except for the case of acne conglobata when it was 4 years after treatment.

## 8 DISCUSSION

The safety profile of combining oral ISO with EBDs for acne management has been studied in several consensus statements, yet comprehensive systematic evaluations of the safety and efficacy of combination therapy remain scarce. Through our systematic review, we identified 16 studies investigating the safety and efficacy of energy‐based modalities in conjunction with ISO for acne treatment. Among them, 141 acne patients undergoing ISO therapy received light therapy, while 464 underwent laser therapy, and 71 underwent radiofrequency treatment.

All included studies suggested that the combination of systemic ISO and energy‐based procedures is well tolerated by most patients, and that the evidence was overall consistent regardless of the different evaluation scales used. Frequently observed side effects include mild transient erythema, discomfort during treatment and dryness of skin. Among the 604 patients reviewed, there were no reports of keloid formation and severe adverse events post‐treatment, with only one instance of blistering observed following IPL treatment and seven cases of acne flare.

Seven studies, including five RCTs, showed that combining treatments with IPL, PDT, PDL, Nd:YAG lasers, Er:glass, and CO_2_ lasers yielded significantly better outcomes in reducing acne severity, lesion counts, and erythema compared to ISO monotherapy.^24^, ^29^, ^35^, ^38^, ^40^ Notably, patients undergoing combination therapy reported higher satisfaction rates in five studies. These findings show the potential clinical benefits of concomitant use of ISO with other EBDs in the early intervention of acne.

ISO remains the choice of first‐line for treating moderate to severe acne.^41^, ^42^ The undesirable consequence of acne scarring underscores the importance of early intervention. Timely intervention of acne scars is critical.^12^ Karmisholt et al. systematically reviewed the efficacy of laser interventions, such as PDL, KTP, Er:YAG, and fractional CO_2_ laser, on acne scars, suggesting that early laser treatment during the inflammation, proliferation, or remodeling phases has the potential to reduce the formation of scar.^43^

However, challenges such as the need for prolonged treatment and side effects including dryness and teratogenicity often result in poor patient compliance.^44^ The debate over postponing energy‐based procedures until 6 months after ISO is due to concerns about photosensitivity and keloid formation and delayed wound healing.^14^, ^17^ Despite the controversy regarding a 6‐month interval between oral ISO and procedural interventions, an increasing body of evidence supports the safe and beneficial use of light and laser treatments shortly after or during ISO therapy. For example, PDL, which targets blood vessels within the dermis, can reduce inflammatory processes and collagen production, thus effectively decreasing erythema and improving acne scars.^45^ Additionally, Kim et al. demonstrated that combining ablative CO_2_ laser with ISO significantly reduced the severity of acne scars and side effects were acceptable, with the combination treatment group experiencing only transient erythema and edema.^33^ As the wavelengths of these devices are typically above 400 nm, the risk of inducing photosensitivity reactions is relatively low.^35^ Given that severe acne and acne scarring can also affect mental health and quality of life, early comprehensive interventions is advisable.

However, there are limitations in this review on combining EBDs with oral ISO in acne patients, primarily due to a scarcity of RCTs, and small study sizes, as well as other methodological flaws such a lack of blinding and control groups. We identified six level 2, three level 3, and seven level 4 studies, with the majority being observational and featuring small sample sizes. Inconsistencies in energy‐based device types, parameter settings, ISO dosages, and varied evaluation scales for acne severity and patient satisfaction, making it difficult to establish clear comparisons. Furthermore, most trials only assessed the short‐term effects of the combination therapy, which limits our ability to draw conclusions about the long‐term efficacy and safety of these combination therapies. Further high‐quality RCTs with a larger sample size, using standardized procedures and evaluation scales are required.

The evidences of this systemic review determined that a delay of EBDs in patients on ISO within 6 month is unnecessary. Majority studies suggested that most non‐ablative energy treatments are safe, and the combination therapy can achieve a superior treatment outcome and is a promising early intervention for acne scars. We suggest that instead of withholding procedural interventions for patients on oral ISO, personalized treatment and sun protection after procedure are warranted.

## 9 CONCLUSION

Although we should be cautious when interpreting these findings, existing literatures suggests that there is no sufficient evidence to support postponing the use of EBDs in patients who have recently undergone or are currently receiving ISO treatment. The combination therapy could produce a superior improvement in terms of acne inflammation, and is a promising early intervention approach for acne scarring.

## AUTHOR CONTRIBUTIONS

Yidan Xu contributed to data acquisition, analysis, data interpretation, and manuscript drafting. Hao Wang contributed to data acquisition, research, and data interpretation. Linghong Guo contributed to revising the manuscript for intellectual content. Xiang Wen contributed to supervising the review and revised the manuscript for academic content. Michael R Hamblin critically edited the article for content and presentation.

## FUNDING INFORMATION

National Natural Science Foundation of China (81903226).

## CONFLICT OF INTEREST STATEMENT

All authors have completed and submitted the ICMJE Form for disclosure of potential conflicts of interest, and none were reported.

## ETHICS STATEMENT

No ethical approval was required as this manuscript is a review article with no original research data.

## Data Availability

The data that support the findings of this study are openly available in repository name at DOI.
